# Differential Proteomics Identification of HSP90 as Potential Serum Biomarker in Hepatocellular Carcinoma by Two-dimensional Electrophoresis and Mass Spectrometry

**DOI:** 10.3390/ijms11041423

**Published:** 2010-03-31

**Authors:** Yiyi Sun, Zhihe Zang, Xiaohong Xu, Zhonglin Zhang, Ling Zhong, Wang Zan, Yan Zhao, Lin Sun

**Affiliations:** 1 Chengdu Medical College, Chengdu 610083, Sichuan Province, China; E-Mails: yysun@hotmail.com (Y.S.); xhxu@yahoo.com.cn (X.X.); zhangzl@live.cn (Z.Z); zhongling@163.com (L.Z.); wangzan@hotmail.com (W.Z.); zhaoyan88@live.cn (Y.Z.); 2 West China Hospital, Sichuan University, Chengdu 610041, Sichuan Province, China

**Keywords:** proteomics, hepatocellular carcinoma, serum biomarker

## Abstract

The aim of the current study is to identify the potential biomarkers involved in Hepatocellular carcinoma (HCC) carcinogenesis. A comparative proteomics approach was utilized to identify the differentially expressed proteins in the serum of 10 HCC patients and 10 controls. A total of 12 significantly altered proteins were identified by mass spectrometry. Of the 12 proteins identified, HSP90 was one of the most significantly altered proteins and its over-expression in the serum of 20 HCC patients was confirmed using ELISA analysis. The observations suggest that HSP90 might be a potential biomarker for early diagnosis, prognosis, and monitoring in the therapy of HCC. This work demonstrates that a comprehensive strategy of proteomic identification combined with further validation should be adopted in the field of cancer biomarker discovery.

## 1. Introduction

Hepatocellular carcinoma (HCC) is the most common primary liver cancer, the fifth most common cancer and the third leading cause of cancer-related death in the world [1,2]. The incidence of HCC is rising around the world with few exceptions. There is a distinct geographical variation in the incidence of HCC, with 81% of cases occurring in the developing world and 54% of these occurring in China [3]. Current curative options can be applied to a paucity of patients, and in general, the prognosis of HCC is dismal due to underlying cirrhosis as well as to poor tumor response to chemotherapeutic regimes [4,5]. Currently, there is an urgent need to devise critical tools for the early diagnosis of and the monitoring of disease progression. Among these tools, validated biomarkers are viewed as the most important; therefore there is a critical need to discover new specific biomarkers in HCC.

Proteomic analysis is currently considered to be a powerful tool for global evaluation of protein expression, and proteomics has been widely applied in analysis of diseases, especially in the field of cancer research [6–9]. Biomarker discovery and validation is a central application in current proteomic research to improve the diagnosis, treatment monitoring and prognosis of many kinds of cancer [6–9]. It has been thought that the association between protein alterations and malignancy—analysis of the cancer proteome—could be more informative and advantageous than genomics or transcriptomics alone, because there are also factors relating to molecular changes in translation, post-translational modification and intracellular mislocalization involved in tumor initiation and growth [10,11]. Two-dimensional gel electrophoresis (2-DE) is an established technology for the separation of proteins. Despite a number of limitations, the resolution of 2-DE gels is impressive, rendering this technology still a preferred tool in many proteomics studies [12,13]. 2-DE gels can also give both qualitative and quantitative analysis and has been applied to proteomic studies in several human cancers, such as colorectal cancer, breast cancer, lung cancer, gastric cancer, prostate cancer, pancreatic cancer, *etc*. [14–19].

Plasma/serum peptides or proteins that are biological indicators are known as biomarkers. Serological biomarker detection promises noninvasive and financially reasonable screening for early cancer with a high potential of positive impact on survival and quality of life and therefore the potential to greatly enhance screening acceptance [9,20]. Alphafetoprotein (AFP), the only clinically available serum marker, has been widely used for serological diagnosis of human HCC. However, the measurement of AFP is not an ideal method for screening individuals at risk of developing HCC due to its poor sensitivity and specificity [21–24]. Therefore, the search is still ongoing to improve early and specific detection and disease monitoring. In the present study, 2-DE and MALDI-TOF MS were employed to investigate protein expression alterations between HCC and control serum samples. The aim of our study was to identify novel tumor-associated molecules for potential biomarkers using 2-DE based differential proteomics.

## 2. Materials and Methods

### 2.1. Serum Sample Collection

Blood samples were collected from 20 patients with HCC consulted for medical care without any previous treatment and 20 healthy donors under fasting conditions. Patients were admitted for HCC surgery, and the HCC was confirmed by pathohistology after surgery. The age range of the healthy donors matched that of the patients. Blood drawn from both donors and patients was allowed to coagulate at room temperature for 10 min and then centrifuged at 3,000 g for 10 min. Serum were then stored at −80 °C. Written informed consent of all patients and blood donors was documented, and anonymity was maintained by tracing the patients through their clinical history number. The project was approved by the Scientific and Ethical Committee of the Chendu Medical Collegue, China.

### 2.2. Serum High-Abundance Protein Depletion

Serum samples from 10 HCC patients and 10 healthy controls were processed to deplete the top-6 (albumin, IgG, IgA, antitrypsin, transferrin, and haptoglobin) high abundance proteins using the Agilent Multiple Affinity Removal System (Agilent Technologies, Palo Alto, CA) with previously published protocols [25,26]. Samples were processed according to the manufacturer’s instructions. For each sample, a low abundance fraction was collected, and buffer was exchanged into 10 mM Tris-HCl (pH 7.4) using 5,000 Da molecular weight cut-off spin concentrators (Agilent Technologies, Palo Alto, CA). Protein concentration was measured by bicinchoninic acid (BCA) protein assay.

### 2.3. Two-dimensional Gel Electrophoresis

All reagents and apparatus used have been described in detail elsewhere [27,28]. Two hundred microliters of serum was mixed with 400 μL of ice-cold acetone (−20 °C) and centrifuged at 10,000 g at 4 °C for 10 min. The pellet was mixed with 10 μL of a solution containing 10% sodium dodecyl (SDS) (w/v) and 2.3% dithioerythritol (DTE) (w/v). The sample was heated to 95 °C for 5 min, then diluted to 60 μL with a solution containing 8 M urea, 4% 3-[(3-cholamidopropyl)dimethylammonio]-1-propanesulfonic acid (CHAPS) (w/v), 40 mM Tris, 65 mM DTE, and a trace of bromphenol blue. The whole final diluted CSF sample corresponding to 50 μg was loaded in a cup at the cathodic end of the immobilized pH gradient (IPG) strips. 2-DE was performed as described previously [29–32]. IEF was performed on rehydrated 18 cm Immobiline Drystrip gels, pH 3–10 NL following the manual of Amersham Biosciences. Vertical acrylamide-bisacrylamide gradient gels (9–16% T, 2.6% C) were used for 2D electrophoresis.

### 2.4. Image Analysis

Image analysis was performed using PDQuest 2D-analysis software (Bio-Rad, Hercules, CA). Spot intensity was quantified automatically by calculation of spot volume after normalization of the image by taking the ratio of intensity of one spot to the total spots, and expressed as a fractional intensity. Only those spots with 3.5-fold (t test, P < 0.05) or more changes in expression intensity were selected for MALDI-TOF-MS analysis.

### 2.5. Tryptic Digestion

Protein spots of interest were excised and destained with 25 mM ammonium bicarbonate, 50% ACN. Gels were then dried completely by centrifugal lyophilization. In-gel digestion was performed with 0.01 μg/μL trypsin in 25 mM ammonium bicarbonate for 15 h at 37 °C. Each spot was digested overnight in 12.5 ng/mL trypsin in 0.1 M NH4HCO3. The peptides were extracted three times with 50% ACN, 0.1% TFA. The extracts were pooled and dried completely by centrifugal lyophilization.

### 2.6. Protein Identification by MALDI-TOF

MALDI-TOF MS analysis of the samples was carried out on a mass spectrometer Autoflex (Bruker Daltonics) in a positive ion reflector mode. The ion acceleration voltage was 20 kV. Each spectrum was internally calibrated with the masses of two trypsin autolysis products. For PMF identification, the tryptic peptide mass maps were transferred with the MS BioToolsTM program (Bruker Daltonics) using MASCOT software (Matrix Science). Then the National Center for Biotechnology nonredundant (NCBInr) database was searched with human as the taxonomy. Up to one missed tryptic cleavage was considered and a mass accuracy of 100 ppm was used for all the tryptic-mass searches.

### 2.7. ELISA for HSP90

Human HSP90 ELISA kits (Adlitteram Diagnostic Laboratories) were used according to the manufacturer’s instructions to detect serum HSP90 levels. Briefly, 100 μL of a standard was dispensed into each of eight wells, and 100 μL of specimens, including 20 serum samples of HCC patients and 20 serum samples of normal controls, were dispensed into plate wells. After dispensing 50 μL of enzyme conjugate reagent into each well, the solutions were gently mixed for 15 s. Then, the plate was incubated at 37 °C for 60 min. After removal of the mixture from the incubator, the microtiter wells were rinsed with deionized water and emptied five times. Then, the wells were sharply stricken onto absorbent paper to remove residual water droplets. Subsequently, 50 μL of color A and color B Reagent was added to each well, and the solutions were incubated at 37 °C for 15 min. The reaction was stopped with the addition of 50 μL of stop solution into each well and gently mixed for 30 s. It is important that all the blue color changes completely to yellow in each well and that the optical density is read at 450 nm within 30 min in a microtiter plate reader.

## 3. Results

### 3.1. Differential Protein Expression in HCC Patients Included in the Study

**Figure 1.**
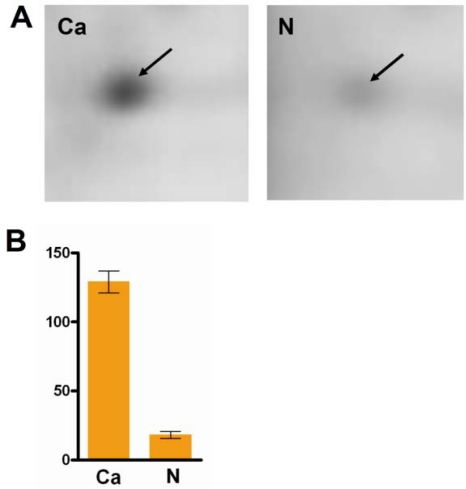
Cropped 2-DE gel images of HSP90 in the serum of hepatocellular carcinoma patients and normal healthy controls. HSP90 was shown the most significant differences in expression between HCC (129.0 ± 4.583) and normal healthy controls (18.33 ± 1.453) (P < 0.0001). It was up-regulated more than 7-fold in the serum of HCC when compared with the normal healthy controls. Ca, carcinoma; N, normal healthy controls.

**Figure 2.**
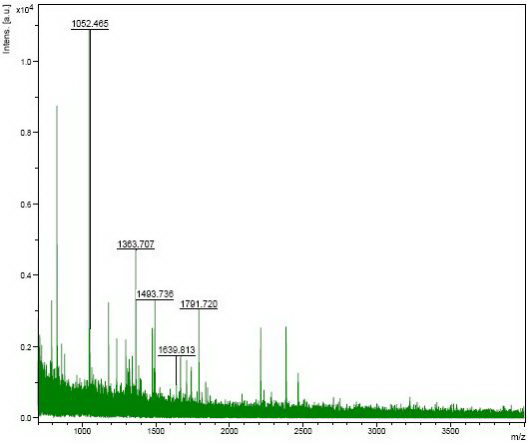
Results of HSP90 as the representative protein identified using MALDI-TOF-MS.

The protein expression profiles in the serum of HCC and healthy controls were obtained by 2-DE. Gel images and representative 2-DE maps were unambiguously matched by the PD-Quest software, and displayed well-resolved and reproducible profiles for both HCC and normal healthy controls. Aprroximately 500–800 protein spots were detected by Coomassie brilliant blue staining in a single 2-DE gel. The quantity of each spot in a gel was normalized as a percentage of the total quantity of all spots in the gel. In comparison with 2-DE patterns, differentially expressed proteins were defined as statistically meaningful on the basis of 3.5-fold up-regulation and 3.5-fold down-regulation in HCC patients compared with healthy controls or more changes in expression intensity (P < 0.05). According to these crtiteria, 35 spots were selected and analyzed using MALDI-TOF-MS. A total of 12 proteins from the 35 spots were identified (Table 1). One of the proteins, HSP90, was found to show the most significant differences in expression between HCC (129.0 ± 4.583) and normal healthy controls (18.33 ± 1.453) (P < 0.0001). HSP90 was up-regulated more than 7-fold in the serum of HCC when compared with the normal healthy controls (Figure 1), and the representative map of mass spectrometry was shown in Figure 2. Redundancy of proteins that appeared in the database under different names and accession numbers was eliminated. If more than one protein was identified in one spot, the single protein member with the highest protein score (top rank) was singled out from the multiprotein family.

### 3.2. Validation of the Significantly Differentially-Expressed Protein HSP90 by ELISA

**Figure 3.**
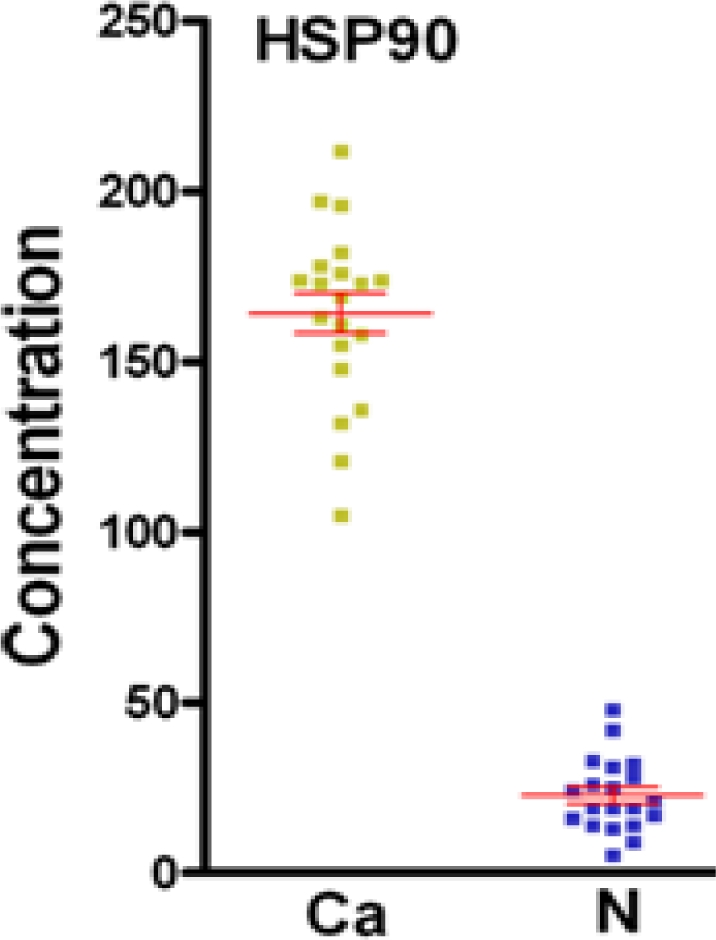
Levels of the potential biomarker HSP90 in the serum of 20 HCC patients and 20 normal healthy controls groups as measured by ELISA assay. The t-test showed that HSP 90 in serum levels of HCC patients (164.7 ± 6.566 ng/mL) were significantly elevated when compared with the healthy controls (22.7 ± 2.728 ng/mL) (P < 0.0001).

Using a commercially available ELISA kit for HSP90 protein, the serum levels of the HSP90 protein was quantified in all 40 individual serum samples (the 20 HCC patients and 20 normal healthy controls) by enzyme-linked immunosorbent assay in order to validate its potential as a candidate biomarker. As shown in Figure 3, the t-test showed that HSP90 in serum levels of HCC patients (164.7 ± 6.566 ng/mL) was significantly elevated when compared with the healthy controls (22.7 ± 2.728 ng/mL) (P < 0.0001), which correlates with the 2-DE results.

## 4. Conclusions

Biomarker searching and tumor profiling in HCC has been intensively carried out in recent years by using DNA microarray technology [33–36]. In general, there have been many discrepancies in the deregulated genes reported by those publications, indicating a high variability in the results depending on the type of platform used (oligos, cDNA, *etc.*) and the type of samples. At the moment, there are no clear candidates for biomarkers of tumor progression or early stages in HCC derived from genomic studies. Because the functional molecules in cells are proteins, proteome analysis is believed to have an advantage over cDNA microarray for clinical use. Proteomics provides the complementary information to that obtained from mRNA profiling by microarray [37,38]. Proteomic researches could lead to the molecular characterization of cellular events associated with cancer progression, cellular signaling, and developmental stages [39,40]. Proteomic researches of clinical cancer samples have led to the identification of cancer-specific protein biomarkers, which could provide a basis for developing new methods for early diagnosis and early detection [41].

In the present study, we compared the global protein profiles between the serum of HCC patients and normal controls using a 2-DE and MALDI-TOF-MS approach. This approach makes no assumptions about known or unknown molecules, allowing the process to be independent of any presupposed hypotheses. A total of 12 differentially expressed proteins were identified, most of which were involved in the biological process, including cell transformation, protein folding, cell proliferation and apoptosis, and so on, which usually play important roles in the malignant initiation and development. Of the 12 identified proteins, seven proteins were up-regulated and five proteins were down-regulated in the serum of HCC. HSP90 shows one of the most significant changes between the serum of HCC and normal controls, and the mass spectrometric identification of HSP90 was also reliable. After showing significant elevation of HSP90 in the serum of HCC patients when compared with the serum of normal healthy controls, the result was validated by ELISA to test the levels of HSP90 in the serum of HCC patients and healthy controls. The results showed that the mean serum level of HSP90 in HCC patients was significant higher than in normal healthy controls, which correlated with the 2-DE results.

Hsp90 is an essential chaperon for function and integrity of a wide range of oncogenic client proteins [42]. Hsp90 is 2-fold to 10-fold overexpressed in tumors, compared with normal tissues [43]. With respect to HCC, Hsp90 overexpression has been described and is associated with a poor prognosis [44]. Recently, several studies have found that the molecular chaperone Hsp90 constitutes a relevant therapeutic target in various types of cancer [45,46]. Oncogenic transcription factors and signaling intermediates have been identified as Hsp90 client proteins, including hypoxia-inducible-factor-1(HIF-1), signal transducer and activator of transcription-3 (STAT3), intracellular kinases (Akt, Erk), and growth-factor receptors [47]. It has been demonstrated that HSP90 controls the folding and activity of numerous bona fide oncoproteins to which malignant cells become addicted, and hence it safeguards the dysregulated expression of these proteins and their mutational status [48]. Hsp90 up-regulation in combination with cyclin-dependent kinase 4 (CDK4) activity, has been predicted to contribute to HCC development [44]. Therefore, HSP90 inhibition is anticipated to surpass targeted therapies that exclusively attack one oncoprotein, the function of which might be compensated via collateral pathways [45–47].

The finding of the current study suggested that HSP90 expression has potential clinical impact. However, the sample size in the current study is relatively small, indicating that the proteomic data need to be confirmed in a study employing a great number of patients and healthy controls in the future. Moreover, more patient-centered and much larger sample numbers are required. Such an approach is in concordance with the conclusions of the Worldwide Strategic Consensus Conference on Biomarker Research; that biomarker research efficiently translates any finds into reduced mortality and morbidity from disease [49–51]. However, biomarkers are known to have the potential to dramatically alter options and strategies of diagnosis and treatment, and consequently a complex process with multiple steps of further analyses is necessary for translating our pilot proteomic observations into clinical applications in the future.

## Figures and Tables

**Table 1.** Differentially expressed proteins identified by MALDI-TOF PMF in the serum of hepatocellular carcinoma and normal healthy controls.

**Spot Number**	**Protein name**	**MW (kDa)**	**pI**	**Fold change (Ca/N)**	**P value (t test)**
1	S-adenosylmethionine synthetase isoform type-1	43.6	5.86	4.21	0.005
2	Glycine N-methyltransferase	32.6	6.58	4.56	0.007
3	Haptoglobin precursor	38.9	6.42	5.10	0.004
4	Serum amyloid P-component precursor	25.4	6.10	3.84	0.018
5	Glyceraldehyde-3-phosphate dehydrogenase	35.9	8.58	5.73	0.003
6	Heat shock protein 90	83.1	4.97	7.04	<0.0001
7	Annexin V	35.8	4.98	3.89	0.012
8	Carbonic anhydrase I	28.9	6.59	−6.73	0.002
9	Beta-galactoside-binding lectin	15.1	5.34	−6.09	<0.0001
10	Vitamin D-binding protein	52.8	5.20	−5.24	0.004
11	Apolipoprotein A-I	28.1	5.30	−3.59	0.017
12	Annexin 4	36.1	5.80	−4.32	0.006
